# Multi-Component Antioxidative System and Robust Carbohydrate Status, the Essence of Plant Arsenic Tolerance

**DOI:** 10.3390/antiox9040283

**Published:** 2020-03-27

**Authors:** Monika Kofroňová, Aneta Hrdinová, Petra Mašková, Jana Tremlová, Petr Soudek, Šárka Petrová, Dominik Pinkas, Helena Lipavská

**Affiliations:** 1 Department of Experimental Plant Biology, Faculty of Science, Charles University, Viničná 5, 2, 128 44 Prague, Czech Republicanaska@centrum.cz (A.H.); lipavska@natur.cuni.cz (H.L.); 2 Faculty of Agrobiology, Food and Natural Resources, Czech University of Life Science, Prague 6, Kamýcká, 961/129 Suchdol, Czech Republic; tremlova@af.czu.cz; 3 Laboratory of Plant Biotechnologies, Institute of Experimental Botany, Academy of Sciences of the Czech Republic, Rozvojová, 313 Prague 6-Lysolaje, Czech Republic; soudek@ueb.cas.cz (P.S.); petrova@ueb.cas.cz (Š.P.); 4 Department of Genetics and Microbiology, Faculty of Science, Charles University, Viničná 5, 2, 128 44 Prague, Czech Republic; dominik.pinkas@natur.cuni.cz

**Keywords:** arsenate, arsenic, arsenite, antioxidant, antioxidant enzyme, saccharides, oxidative stress, ROS, tolerant and sensitive tobacco

## Abstract

Arsenic (As) contaminates the food chain and decreases agricultural production through impairing plants, particularly due to oxidative stress. To better understand the As tolerance mechanisms, two contrasting tobacco genotypes: As-sensitive *Nicotiana sylvestris* and As-tolerant *N.tabacum*, cv. ‘Wisconsin’ were analyzed. The most meaningful differences were found in the carbohydrate status, neglected so far in the As context. In the tolerant genotype, contrary to the sensitive one, net photosynthesis rates and saccharide levels were unaffected by As exposure. Importantly, the total antioxidant capacity was far stronger in the As-tolerant genotype, based on higher antioxidants levels (e.g., phenolics, ascorbate, glutathione) and activities and/or appropriate localizations of antioxidative enzymes, manifested as reverse root/shoot activities in the selected genotypes. Accordingly, malondialdehyde levels, a lipid peroxidation marker, increased only in sensitive tobacco, indicating efficient membrane protection in As-tolerant species. We bring new evidence of the orchestrated action of a broad spectrum of both antioxidant enzymes and molecules essential for As stress coping. For the first time, we propose robust carbohydrate metabolism based on undisturbed photosynthesis to be crucial not only for subsidizing C and energy for defense but also for participating in direct reactive oxygen species (ROS) quenching. The collected data and suggestions can serve as a basis for the selection of plant As phytoremediators or for targeted breeding of tolerant crops.

## 1. Introduction

Arsenic, a toxic metalloid, is a non-essential element and group I carcinogen. Its accumulation in soil and water occurs due to anthropogenic activities and natural processes [1]. It exists in many chemical forms with varying degrees of mobility, bioavailability, and toxicity [2]. The proportion of individual forms is influenced by soil structural and chemical characteristics and therefore soil characteristics are important for As uptake and distribution within the plant body [3]. Arsenic affects basal cellular metabolism [4] and its presence in the environment could hamper plant growth and induce a biomass reduction [5,6,7,8]. 

Arsenic intake by plants can hardly be down regulated because it is often mediated by essential element transporters. Under oxidative conditions, arsenate (As^V^) prevails in soil and, as a phosphate analogue, it enters plant cells via phosphate transporters [9], e.g., AtPht1;1 and AtPht1;4 in *Arabidopsis* [10] or OsPht1;1 in rice [11]. Under reducing conditions, arsenite (As^III^) is dominant and its absorption is enabled by nodulin 26-like intrinsic proteins, a class of aquaporins [12]. Due to the chemical similarity of arsenite with silicic acid, it can also enter plant cells by Si transporters that have been found, e.g., in rice [12]. Once inside the cell, most of As^V^ is reduced to As^III^ as only the trivalent form can undergo detoxification. As^V^ interferes with phosphate metabolism, and As^III^ reacts with proteins´ sulfhydryl groups (–SH) and thus damages cellular functions [13]. Very frequent negative effects of heavy metals, including metalloid arsenic, result in a drop of the photosynthetic rate [14,15,16]. The severity of photosynthetic apparatus damage is related chiefly to the dosage of the arsenic treatment and applied As form. In primary photosynthetic reactions, arsenic affects chlorophylls’ and carotenoids’ integrity, thus impairing the activity of photosystems’ antenna complexes as well as the electron transport chain, which results in reduced ATP and NADPH syntheses and/or increased energy dissipation via fluorescence or as heat [17]. The latest studies published mention the negative photosynthesis response upon arsenic, e.g., in tobacco-related potato [18] or tomato [16], but also unrelated *Artemisia annua* [14,15] or *Ricinus* [19]. These results, although often accompanied by data showing growth retardation or even plant death, are only rarely combined with carbohydrate status determination as also reviewed by Abbas et al. [20] and Kofroňová et al. [21]. However, different environmental stresses adversely affect carbohydrate metabolism, thus having a far-reaching impact on plants [22,23]. Carbohydrate levels and spectra are of utmost importance for it does not mean only the availability of C and an energy source for metabolism support (including the stress one). Carbohydrates also function as important indicators of the energy status and plant growth potential (i.e., signaling system) and therefore they are very important for data interpretation.

In the context of arsenic stress, it is important to note that it induces the production of reactive oxygen species (ROS) [16,24,25,26]. To protect themselves, plants have developed sophisticated defensive mechanisms, including the action of antioxidant enzymes (superoxide dismutase, catalase, peroxidases like ascorbate peroxidase and glutathione-S-transferase) and antioxidant molecules (e.g., phenolic compounds, ascorbate, glutathione, and proline) [27]. Among the most important free-radical quenchers, carbohydrates, particularly sucrose, raffinose family oligosaccharides, or fructans, can also eliminate the most reactive ROS, hydroxyl radical (OH·), which cannot be directly quenched by antioxidant enzymes [28]. A specific advantage of carbohydrates, versus other antioxidant molecules, lies in their ubiquity. Plant antioxidative defense under As stress, including the notion of carbohydrate participation, has recently been reviewed by Kofroňová et al. [21]. Carbohydrates, besides their ROS quenching capacity, act in stress defense indirectly via the provision of energy and carbon sources for defense molecule syntheses, including antioxidant enzymes [29]. Moreover, sucrose together with glucose are capable of controlling gene expression connected with stress resistance (e.g., [30]). Glucose accumulation alleviates the damaging effects of stress by enhancing the production of antioxidants and compounds, which act as osmotica by maintaining the water potential inside the cell, pH homeostasis regulator, and reduce membrane permeability during stress [31]. Signaling mediated by sucrose appears to be essential for the regulation of anthocyanin synthesis [32]. Besides anthocyanin, the synthesis of other antioxidant molecules, e.g., ascorbate or phenolic compounds, can also be controlled by sucrose signaling [33]. Saccharides are, besides what has already been said, involved in pathways producing NADPH, which is necessary for the activity of monodehydroascorbate reductase and glutathione reductase [34], enzymes active in oxidative stress defense. Unfortunately, the As effect on carbohydrate status needs further clarification, as not many of the studies devoted to the As influence on saccharide metabolism used various plant species, a diverse experimental design, or material of different plant ages; moreover, these studies led to contradictory results (decreased/increased or even fluctuating saccharide levels, e.g., in rice [35], bean [36], wheat [37], potato [18], or tomato [16]. Importantly, As-tolerant fern *Pityrogramma calomelanos* exhibited relative stable carbohydrate levels upon arsenic stress [38]. 

In this study, we tested the following hypothesis: A highly effective multicomponent antioxidative system supported by stable carbohydrate metabolism arising from undisturbed photosynthesis is the inevitable prerequisite for strong As stress tolerance in plants. To support or reject this idea, we performed a complex analysis of the growth characteristics, photosynthesis vulnerability, oxidative stress levels, and components of the antioxidant system involved in stress buffering in arsenic-exposed sensitive and tolerant tobacco species with a focus on carbohydrates. The results indicate that the tolerant genotype exhibits a better ability to alleviate oxidative stress and activate antioxidant components at the appropriate location. The tolerance also lies in stable levels of carbohydrates, possible ROS quenchers, as well as energy sources for stress amelioration. 

## 2. Materials and Methods

### 2.1. Selection and Cultivation of Plant Material

Out of seven tobacco genotypes (*Nicotiana sylvestris, Nicotiana glutinosa* and five cultivars of *Nicotiana tabacum,* cv. La Burley, Samsun, Petit Havana, Bright yellow, Wisconsin, the seeds from Institute of Biophysics, the Czech Academy of Sciences), arsenic tolerant/sensitive genotypes were selected based on the growth results of one-week-old plant seedlings cultivated in vitro for 14 days on 30 and 60 µg l^−1^ arsenate. *N.tabacum* cv. Wisconsin (best growing) and *N. sylvestris* (worst growing) were chosen as the tolerant and sensitive genotype, respectively, for further analyses.

Growth conditions: The seeds were sown on a sterilized horticultural substrate and two-week-old uniformly sized plantlets were transferred to pots (one plant per pot) with substrate (perlite and sand, 1:1). Ten days after transfer, the plants started to be regularly watered with Na_2_HAsO_4_.7H_2_O (10 or 30 µg l^−1^) containing or As-free Hoagland solutions. The experiments were repeated 4 times in June and July (2014–2017). Plant analyses were performed after 7 weeks of arsenic exposure with 3–5 biological replications per treatment. For biomass determination, fresh or freeze-dried roots and shoots were used. 

### 2.2. Arsenic Content Determination

For the determination of As levels and species, samples of 1 ± 0.1 g of dried and powdered plant material were used. First, 10 mL of 0.02 M NH_4_H_2_PO_4_ (pH 6.0) was added and the suspension was transferred to a tube. The tubes were fastened to a cross-shaped rotor and turned top-over-bottom at 2668 x g for 14 h. Then, the suspension was centrifuged for 10 min at 3000 rpm and filtered through syringe cellulose-nitrate ester filters (0.22 µm). Individual As compounds were determined by high-performance liquid chromatography (HPLC 1260 series, Agilent Technologies) equipped with an anion-exchange column (PRP-X100, 150 × 4.6 mm with 5μm particles, Hamilton); isocratic elution, RT, mobile phase: 0.02 M NH_4_H_2_PO_4_ (pH 6.0), flow rate: 1.5 mL min^−1^. An element-selective detector composed of Agilent inductively coupled plasma mass spectrometry (ICPMS) 7700x equipped with a helium collision cell and a quadrupole and outlet was connected with a PEEK capillary (0.125 mm i.d.) to the nebulizer of the ICPMS system. The intensity of ions at *m/z* 75 (^75^As and ^40^Ar^35^Cl) and also potential argon chloride (^40^Ar^37^Cl) interferences at *m/z* 77 were monitored. For details, see [39].

### 2.3. Photosynthetic Characteristics Determinations

*Photosynthesis rate and stomatal conductance:* The net photosynthetic rate (P_n_) and stomatal conductance (g_s_) of intact leaves were determined using an LI-6400 portable gas analyzer (LI-COR, Lincoln, NE) at 25 °C, 600 µg l^−1^ of CO_2_, and relative air humidity adjusted to at least 60%. The measured data were collected at 1-min intervals. The protocol of changing irradiation consisted of a 5-min initial period (at irradiation 200 µmol m^−2^ s^−1^) followed by a 10-min dark phase and measurements under changing irradiations in the following sequence: 100, 300, 500, 700, 900, 1100, and 200 µmol m^−2^ s^−1^ (5-min interval for each). 

*Fast fluorescence kinetics* was determined on dark-adapted intact leaves (leaves to be measured were covered with aluminum foil to ensure full oxidation of photosystem reaction centers for 1 h). Using a pocket fluorimeter FluorPen 2 (PSI, Photon system instrument), the rapid onset of fluorescence induced by the blue light pulse was measured. The measurements were repeated at 3 places in the worksheet [40]. The maximum quantum yield of the primary PSII photochemistry was calculated as (Fm-Fo)/Fm, where Fm is the maximum value of fluorescence under saturating irradiance and Fo is the initial value of the fluorescence.

*Photosynthesis pigment content determination:* Cut-outs from leaves with a 5mm diameter were plunged in N,N-dimethylformamide to extract the photosynthetic pigments (chlorophyll a and b and total carotenoids). The concentration of pigments was than determined spectrophotometrically (Evolution 201, programme Thermo insight); absorbances at 480, 647, 664, and 750 nm were followed; and pigment contents calculated according to Equations (1), (2), and (3):

Ch_a_ = 11,65 * A_664_ – 2,69 * A_647_ (μg mL^−1^), (1)

Ch_b_ = 20,81 * A_647_ – 4,53 * A_664_ (μg mL^−1^), (2)

C_(x + c)_ = (1000 * A_480_ – 0,89 C_a_ – 52,02 * C_b_) / 245 (μg mL^−1^), (3)

where Ch_a_ is chlorophyll a; Ch_b_ is chlorophyll b; and C_(x + c)_ is the total concentration of carotenoids (xantophylls and carotens) reported by Wellburn [41].

### 2.4. Oxidative Stress Level Determination

*Reactive oxygen species (ROS) content determination:* Roots and leaves (0.1 g) crushed in liquid nitrogen were mixed with 0.5 mL of 20 mM phosphate buffer (pH 6.4) and the suspension centrifuged at 4000× *g* (10 min, 4 °C). The ROS content was measured as an increase in the fluorescence of fluorescein-based dye using a Fluoromax 3 instrument with a Micromax microplate reader (Jobin-Yvon Horiba, Bensheim, Germany). Detection principle: In the presence of ROS, the non-fluorescent 2’,7’-dichlorodihydrofluorescein diacetate (H_2_DCFDA) (Sigma) is cleaved and oxidized to the highly fluorescent 2’,7’-dichlorofluorescein (DCF). The excitation and emission wavelengths were set to 485 and 522 nm with 490SP and 510LP filters, respectively, in [42]. 

*Malondialdehyde (MDA) level determination* was performed spectrophotometrically following Hodges et al. [43] with some modifications. Fresh samples from each treatment (0.1 mg) were homogenized in 2 mL of 80% ethanol (v/v) and centrifuged at 19,000× *g* for 20 min at 4 °C. The obtained supernatant was mixed with 700 µL of 20% TCA (trichloroacetic acid, v/v) containing 0.5% TBA (2-thiobarbituric acid) and then heated in boiling water for 30 min, and then cooled rapidly in an ice bath. The samples were centrifuged again at 10,000× *g* for 10 min. The amount of MDA was then determined spectrophotometrically (Evolution 201, programme Thermo insight) and calculated from the difference of the absorbances at 440, 532, and 600 nm according to Equations (4), (5), and (6):

C(_MDA)_ = [(A – B)/ ε], (4)

A = [(A_532(+TBA)_ – A_600(+TBA)_) – (A_532 (-TBA)_ – A_600(-TBA)_)], (5)

B = [(A_440(+TBA)_ – A_600(+TBA)_) * 0,0571], (6)

where: ε = corrected extinction coefficient of MDA (157 mM^−1^ cm^−1^); A_532(+TBA)_ – A_600(+TBA)_ = absorbance of TBA-MDA complexes at 532 nm corrected for non-specific absorbance at 600 nm; and A_532 (-TBA)_ – A_600(-TBA)_ = absorbance of compounds in extract-solution without TBA at 532 nm corrected for non-specific absorbance at 600 nm; [(A_440(+TBA)_ – A_600(+TBA)_) * 0,0571] = correction for nonspecific TBA-sugar complexes according to Hodges et al. [43].

### 2.5. Antioxidant Enzymes Activities

*Protein extraction*: Frozen samples (3 g) were ground in liquid nitrogen with 30 mL of phosphate buffer (0.1 M potassium phosphate, 5 mM EDTA (ethylenediaminetetraacetic acid), 1% polyvinylpolypyrrolidone (w/v), 1% Nonidet P40 (Sigma, Germany), 5 mM dithiothreitol, pH 7.8). The mixture was centrifuged at 24,328× *g* at 4 °C for 30 min. The supernatants were stored at −80 °C. The antioxidant enzyme activities in the protein extracts were determined by a UV-VIS spectrophotometer. The activities were related to the total protein content detected using the Bradford method based on bovine serum albumin standard [44] according to the following Equation (7):

(7) $$specific\;activity=\left(\left(\frac{\left(\frac{\Delta A}{\Delta t\left(sample\right)}-\frac{\Delta A}{\Delta t\left(blank\right)}\right).\;V\left(all\right)}{\varepsilon .d.V\left(enzyme\right)}\right).\;1000\right)/C\left(Bradford\right),$$

where: $\frac{\Delta A}{\Delta t(\mathrm{sample})}$ = change in absorbance over time of sample; $\frac{\Delta A}{\Delta t\left(blank\right)}$ = change in absorbance over time of blank; V(all) = volume of cuvette; V(enzyme) = volume of buffer; ε = extinction coefficient; d = optical path length; and C(Bradford) = protein content in sample. *Glutathione-S-transferase (GST) activity measurement*: GST activity was determined after the reaction of GST with CDNB (1-chloro-2,4-dinitrobenzene) in 0.1 M potassium phosphate buffer, pH 6.4 (reaction mixture: 1 mM GSH, 1mM CDNB). The change in absorbance at 340 nm per 5 min was measured at 25 °C in a volume of 190 µL. The enzyme activity was calculated using an extinction coefficient of 9.6 mM^−1^ cm^−1^ [45].

*Peroxidase (POX) activity measurement:* The POX activity in the protein extract was determined after the reaction of 100 mM ABTS (2,2′-azino-bis(3-ethylbenzothiazoline-6-sulphonic acid)) as a substrate and 4.5 mM H_2_O_2_ in 50mM potassium phosphate buffer (pH 7.0). The change in absorbance at 414 nm per 5 min was measured at 25 °C in a volume of 200 µL. The enzyme activity was calculated using an extinction coefficient of 31.1 mM^−1^ cm^−1^ [46]. 

*Catalase activity measurement:* Catalase activity was assayed according to a modified procedure of Beers and Sizer [47]. The mixture contained 200 mM H_2_O_2_ as a substrate and 100 mM KH_2_PO_4_ buffer (pH 7.0). The decomposition of H_2_O_2_ was followed at 240 nm in a volume of 150 µL (extinction coefficient of 0.036 mM^−1^ cm^−1^). 

*Ascorbate peroxidase (APX) measurement:* APX was determined by a modified method of Nakano and Asada [48]. The reaction mixture contained 55.56 mM KH_2_PO_4_ buffer (pH 7.0), 60 mM ascorbate, and 3% H_2_O_2_ (v/v). APX activity was calculated using an extinction coefficient of 2.8 mM^−1^ cm^−1^ at 290 nm in a volume of 200 µL.

### 2.6. Antioxidant Compounds Determinations

*Total phenolic compounds determination:* The total phenolic compounds content of the samples was determined using the Folin–Ciocalteu method, as described by Singleton et al. [49]. Fresh biomass (0.1 mg) from each treatment was homogenized, and the samples were extracted in 4 mL of 10% ethanol (v/v). The calibration curve was determined using gallic acid as a standard in the concentration range 50 to 500 mg l^−1^. The results were expressed as gallic acid equivalents spectrophotometrically at 750 nm.

*Anthocyanins content determination:* Anthocyanins were determined in leaves according to Mancinelli et al. [50]. For extraction, approximately 100-mg dry weight samples were boiled with 0.5 mL acid methanol (pH=1; 0.2 mL concentrated H_2_SO_4_, 9.8 mL CH_3_OH) for 1 min at 85 °C. The samples were placed in the dark at 4 °C. After 24 h of extraction, the samples were centrifuged for 10 min and supernatants were transferred to fresh tubes. The amounts of anthocyanins were measured spectrophotometrically at 530 and 657 nm. The concentrations of anthocyanins were quantified according to the formula A_530_-0.33*A_657_.

*Glutathione level determination:* The glutathione contents were determined according to Griffith [51] with some modifications. Samples of approximately 100 mg of fresh mass were homogenized and mixed with 100 µL of MiliQ H_2_O. The oxidized (GSSG) and reduced (GSH) glutathione contents were determined using the kit: BIOXYTECH^®^GSH/GSSG-412TM, Oxis International, Inc. The contents of GSH and GSSG were determined using calibration curves and the results were expressed in µmol GSH g^−1^ fresh weight. After NADPH addition, the absorbance was measured for 3 min at 412 nm, according to the manufacturer’s instructions.

*Ascorbic acid determination:* Root and leaf samples of approximately 40 mg of fresh mass were homogenized in liquid nitrogen. Extraction and analysis were performed using an Ascorbic Acid Assay Kit II (Sigma-Aldrich) according to the manufacturer’s instructions. Ascorbic acid concentration was determined colorimetrically at 593 nm using the Ferric Reducing/Antioxidant and Ascorbic Acid (FRASC) assay, where Fe^3+^ is reduced to Fe^2+^ by antioxidants present in the sample. Parallel samples with ascorbate oxidase allow measurement of the ascorbate concentration.

### 2.7. Carbohydrate Content Determination 

The samples (100-200 mg) were immediately frozen in liquid nitrogen, then freeze-dried, and their dry weight was determined. For extraction, the samples were boiled with 0.5 mL of 80% methanol (v/v) at 75 °C for 15 min, the solvent was vacuum-evaporated, and the residue was resuspended in Milli-Q ultrapure water (Millipore). The content of non-structural soluble carbohydrates was determined using high performance liquid chromatography (HPLC, flow rate 0.5 mL min^−1^, 80 °C) with refractometric detection (refractive index range 1–1.75; refractometer Shodex RI-71; Spectra Physics—Newport Corporation, Irvine, CA, USA), column: IEX Ca^2+^ (Shodex). The starch in the pellets after the extraction of soluble carbohydrates was hydrolyzed by α-amylase (Fluka, 30U) and amyloglucosidase (Sigma, 60U) in 0.1 M Na-acetate buffer (pH 4.5), and the glucose content was measured by HPLC. The data were evaluated using Clarity 7.2 software (DataApex). 

### 2.8. Statistical Analysis

Values are presented as means of 5-10 independent biological replications ± standard deviation. For comparison of individual arsenic treatments versus the control, one-way analysis of variance (ANOVA) and multiple comparison Dunnett’s post-hoc test (two-Sided versus control) (NCSS 9.0 software, LCC Kaysville) were used. The sample number (n) represents the number of biological replicates per treatment. Individual As treatments significantly different from the control were marked above the column as *** (*p* < 0.001), ** (*p* < 0.01), * (*p* < 0.05), or (*) (*p* < 0.1).

## 3. Results

### 3.1. Arsenic Content and Effect on Biomass Accumulation in Tobacco Roots and Leaves 

**Figure 1:**
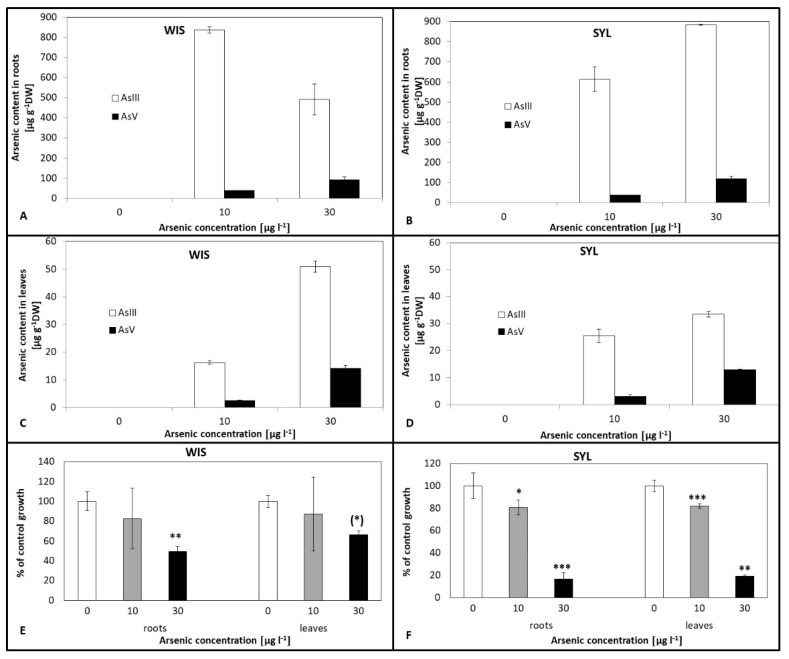
Arsenic contents and the growth response to arsenic treatments in two contrasting tobacco genotypes. (**A**,**C**,**E**) Tolerant *N.t.* cv. Wisconsin; (**B**,**D**,**F**) Sensitive *N. sylvestris*. 0, 10, and 30 µg l^−1^ arsenate concentrations. Bars indicate standard deviations. Stars mean statistically significant differences from the control at the level α = 0.1 ((*)), 0.05(*), 0.01 (**), 0.001(***), n = 5.

Both genotypes accumulated more arsenite than arsenate (Figure 1A–D). Leaf contents of both As forms were one order lower than root ones. In *Nicotiana sylvestris* (SYL) (Figure 1B,D), the leaf and root contents of both As forms increased with an increasing medium As concentration. Interestingly, in *Nicotiana tabacum*, cv. Wisconsin (denoted as WIS) roots, arsenite decreased, but the arsenate content increased (Figure 1A) with increased As exposure, although in WIS leaves, both As forms increased with an increasing As dose (Figure 1C). In all control samples, there was a negligible amount of both forms of As. 

The tolerant tobacco WIS grew in the presence of arsenate much better (Figure 1E) than the sensitive SYL (Figure 1F). In WIS, there was a decrease of root growth with an increasing As dose, though this was only significant for the higher As treatment. On the contrary, in SYL, there was a significant reduction of leaf and root growth already at 10 µg l^−1^ arsenate and a dramatic decrease to less than one fifth in the higher arsenic treatment. 

### 3.2. Effect of Arsenic on Photosynthetic Characteristics 

**Figure 2:**
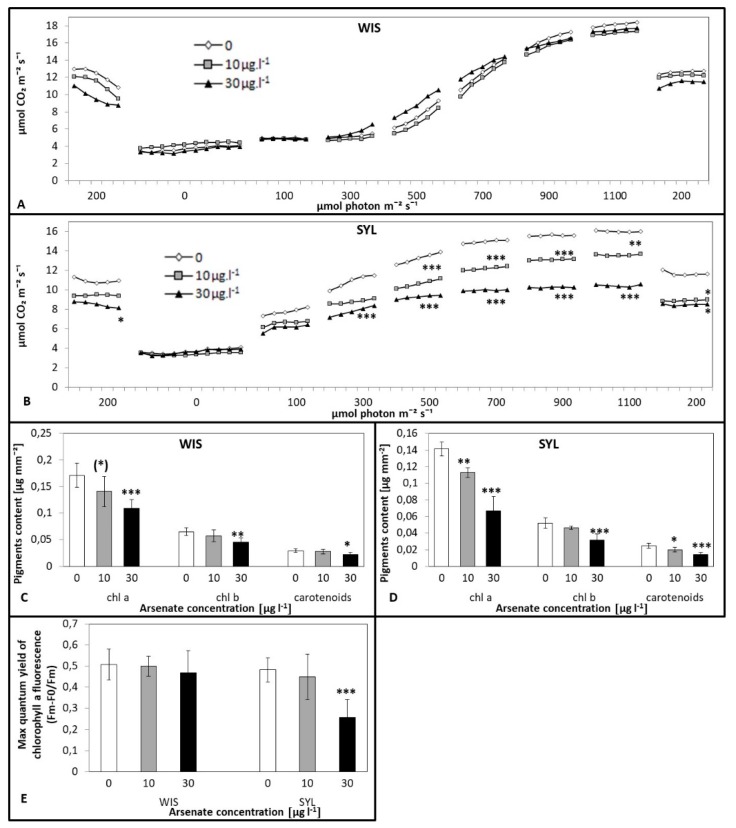
Effects of arsenic on the net photosynthesis rate under different irradiances (**A**,**B**), pigments contents (**C**,**D**), and chlorophyll a fluorescence (**E**) in two contrasting tobacco genotypes (**A**,**C**) tolerant *N.tabacum* cv.Wisconsin; (**B**,**D**) sensitive *N. sylvestris*). 0, 10, and 30 µg l^−1^ arsenate concentrations. Bars indicate standard deviations. Stars mean statistically significant differences from control at the level α = 0.1 ((*)), 0.05(*), 0.01 (**) or 0.001(***), n = 5.

In the tolerant WIS, the net photosynthesis rate response to changing irradiance did not differ significantly between control and arsenic treatments (Figure 2A). In the sensitive SYL, however, the photosynthesis rate decreased in an As dose-dependent manner; the decline was significant for both As doses at irradiances of 500 to 1100 µmol m^−2^ s^−1^ (Figure 2B). 

As regards leaf stomata conductance (GS), the tolerant WIS exhibited a slight tendency to reduced conductance only under the stronger arsenic treatment at higher irradiances. Sensitive SYL, however, exhibited a tendency to decrease at both As treatments, with a drop of 30 µg l^−1^ at higher irradiances (700–1100 µmol photon m^−2^ s^−1^; Figure S1). 

Plastid pigments contents (chlorophylls a and b and carotenoids, Figure 2C,D) decreased with an increasing arsenic dose in both genotypes. For sensitive SYL, the decrease was significant even at 10 µg l^−1^ (except for chlorophyll b). In the sensitive SYL, the above-mentioned chlorophyll content decrease was accompanied by a decrease in the maximum fluorescence efficiency; at the higher As concentration, this was about one half of the control. In the tolerant WIS, however, the fluorescence efficiency was not influenced (Figure 2E).

### 3.3. ROS Content and Lipid Peroxidation under Arsenic Exposure

**Figure 3:**
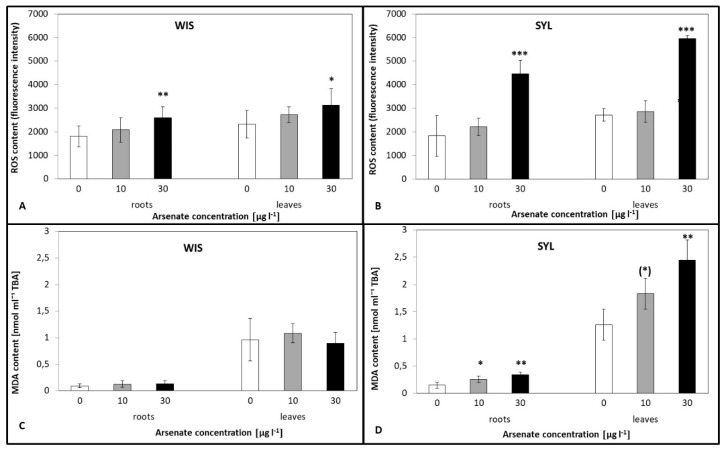
Effect of arsenic on the ROS (**A**,**B**) and malondialdehyde (MDA) (**C**,**D**) contents in two contrasting tobacco genotypes ((**A**,**C**) tolerant *N.tabacum*, cv.Wisconsin; (**B**,**D**) sensitive *N. sylvestris*). 0, 10, and 30 µg l^−1^ arsenate treatments. Bars indicate standard deviations. Stars mean statistically significant differences from control at the level α = 0.1 ((*)), 0.05(*), 0.01 (**) or 0.001(***), n = 5–9.

In As-treated WIS roots and leaves, there was a mild increase in reactive oxygen species (ROS) contents, which was only significant at 30 µg l^−1^ (Figure 3A). In the sensitive SYL, however, there was a statistically significant two-fold increase of ROS at the higher As concentration (Figure 3B), both in leaves and roots. Further, malondialdehyde (MDA) levels were comparable in As-treated WIS leaves and roots (Figure 3C) though the sensitive tobacco SYL exhibited a gradual statistically significant increase of MDA with the increasing As treatment (Figure 3D).

### 3.4. Effects of Arsenic on Antioxidant Enzymes Activities 

**Figure 4:**
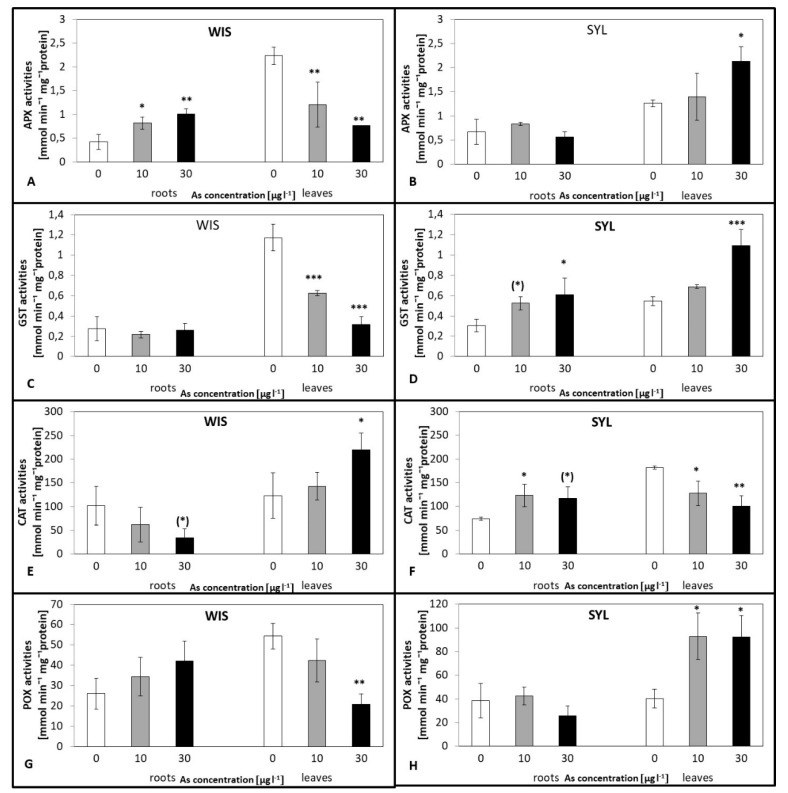
Effect of arsenic on the antioxidant enzymes activities in two contrasting tobacco genotypes ((**A**,**C**,**E**,**G**) tolerant *N.tabacum*, cv.Wisconsin, (**B**,**D**,**F**,**H**) sensitive *N. sylvestris*). 0, 10, and 30 µg l^−1^ arsenate treatments. APX—ascorbate peroxidase (**A**,**B**), GST—glutathione-S-transferase (**C**,**D**), CAT—catalase (**E**,**F**), POX—peroxidase (**G**,**H**). Bars indicate standard deviations. Stars mean statistically significant differences from control at the level α = 0.1 ((*)), 0.05(*), 0.01 (**) or 0.001(***), n = 5.

As regards antioxidant enzyme activities, the genotypes under study exhibited different or even opposite reactions (Figure 4). In tolerant WIS, ascorbate peroxidase (APX) and glutathione-S-transferase (GST) had nearly identical responses in the leaves: A dramatic gradual decrease that was significant for both arsenate treatments (Figure 4A,C). A similar pattern was also found in WIS leaf peroxidase (POX), though this was only significant at 30 µg l^−1^ (Figure 4G). As regards the activities in WIS roots, oppositely, there was a gradual significant increase for APX activity while no significant activity changes for GST and POX were found (Figure 4A,C,G). A reverse response was exhibited by catalase (CAT), where in WIS leaves, the activities gradually increased with increasing As while in roots, they decreased (both significant at 30 µg l^−1^) (Figure 4E). 

Interestingly, in the leaves and roots of sensitive SYL, quite the opposite pattern was determined nearly for all followed enzyme activities (Figure 4B,D,F,H). Increased activities were apparent in the SYL leaves for all enzymes except CAT, where, on the contrary, activities gradually decreased (Figure 4B,D,F,H). Importantly, the changes in APX and GST were statistically significant at higher As treatments. For POX and CAT, significant differences were also found at 10 µg l^−1^. In As-treated SYL roots, there was a significant increase in GST and CAT, but no changes in APX and POX activities (Figure 4B,D,F,H).

### 3.5. Effects of Arsenic on Antioxidant Molecules Levels 

**Figure 5:**
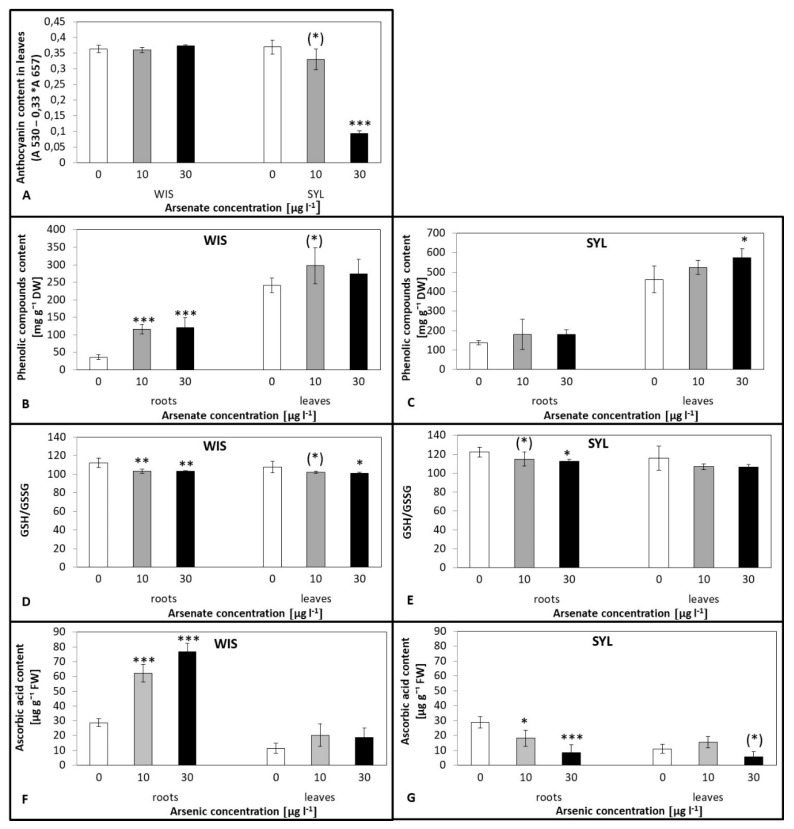
Effect of arsenic on anthocyanin (**A**) and phenolics contents (**B**,**C**), GSH/GSSG - glutathione forms ratio (**D**,**E**), and ascorbate contents (**F**,**G**) in two contrasting tobacco genotypes ((**A**,**B**,**D**,**F**) tolerant *N.tabacum* cv.Wisconsin; (**A**,**C**,**E**,**G**) sensitive *N. sylvestris*). 0, 10, and 30 µg l^−1^ arsenate treatments. Bars indicate standard deviations. Stars mean statistically significant differences from control at the level α = 0.1 ((*)), 0.05(*), 0.01 (**) or 0.001(***), n = 4–5.

The levels of WIS leaf anthocyanins remained stable even under the higher As treatment while the sensitive SYL had a sharp decline at 30 µg l^−1^ (Figure 5A). In the leaves of both genotypes, the total phenolic compound contents tended to increase, though this was only significant for SYL and the higher As dose (Figure 5B,C). In the roots, however, phenolics significantly increased for both As treatments in tolerant WIS, whereas in SYL no response appeared (Figure 5B,C). No prominent changes in root proline contents were detected regardless of the As dose or genotype (Figure S2). In leaves, however, an increasing trend was found in both genotypes at 30 µg l^−1^, though a significant 1.5-fold increase was found only in WIS.

The ratios between reduced and oxidized glutathione (glutathione:glutathione disulphide; GSH:GSSG) showed a trend to gradual decreases in both arsenic treatments and both genotypes (Figure 5D,E), significant in WIS leaves and roots, while in SYL, the decrease appeared solely in roots of the higher As dose but less pronounced than in tolerant WIS. As regards the total glutathione contents in the genotypes under study, in tolerant WIS roots, significantly higher levels were detected under both As treatments, though no difference was found in this parameter in WIS leaves. In sensitive SYL, only higher As exposure resulted in a significant increase solely in the leaves (Figure S3). 

In tolerant WIS, there was a 2.5-fold increase in the ascorbate contents in roots induced by both As treatments (significant at *p* < 0.001). On the contrary, in sensitive SYL, the only change induced by As treatment was a gradual decrease in roots, significant at both 10 and 30 µg l^−1^ (Figure 5F,G). However, in the leaves of both genotypes, no changes in ascorbate levels were detected.

### 3.6. Effects of Arsenic on the Carbohydrate Status 

**Figure 6:**
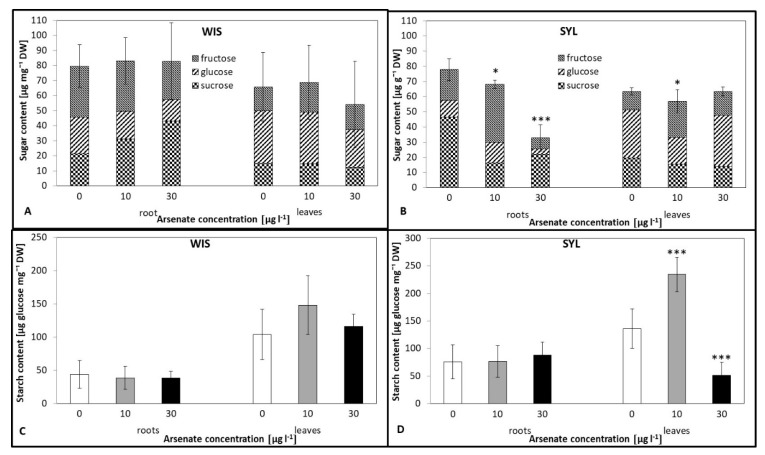
Effects of arsenic on soluble carbohydrate (**A**,**B**) and starch (**C**,**D**) contents in two contrasting tobacco genotypes (**A**,**C**) tolerant *N.tabacum* cv. Wisconsin; (**B**,**D**) sensitive *N. sylvestris*; (**C**,**D**) amount of glucose after enzymatic starch degradation). 0, 10, and 30 µg l^−1^ arsenic treatments. Bars indicate standard deviations. Stars mean statistically significant differences from control at the level α = 0.1 ((*)), 0.05(*), 0.01 (**), or 0.001(***), n = 10.

Analysis of the leaf carbohydrate status revealed no differences between control and As-treated variants for both genotypes, except for a decrease at 10 µg l^−1^ As in SYL (Figure 6A,B). Roots, however, behaved differently. While the tolerant WIS exhibited no significant changes, the sensitive SYL showed a gradual and significant decrease of carbohydrate levels with increasing As stress. On the other hand, as opposed to WIS, where starch levels (expressed as glucose units arisen from starch enzymatic splitting) did not change much in both, the roots and leaves (Figure 6C), the sensitive SYL starch amounts were unchanged only in the roots (Figure 6D) while in leaves, this increased significantly at 10 µg l^−1^ but dropped dramatically to less than a half at 30 µg l^−1^ arsenate.

## 4. Discussion

Elevated arsenic contents in water and soil in many areas are of serious environmental and human health concerns [52]. Most reports demonstrate remarkable As-induced stress symptoms on various levels and numerous physiological responses, leading to growth and developmental changes in plants [7,8,53]. 

### 4.1. Plant Growth and Photosynthesis Under As Stress

In our study, arsenic toxicity resulted in an evident biomass reduction in both relatively tolerant *Nicotiana tabacum* cv. Wisconsin (WIS) and sensitive *Nicotiana sylvestris* (SYL), though the drop was much more serious in SYL and observed at a lower arsenic concentration (Figure 1E,F). Both genotypes accumulated up to two orders more arsenic in roots than in leaves, mainly as As^III^. Plant fitness can be affected by arsenic through interference with photosynthesis [54]. According to Chandrakar et al. [55], As toxicity includes reduced growth and biomass accumulation, leaf gas exchange, chlorophyll synthesis, and thereby affects the photosynthesis rate. Generally, under arsenic treatment, photosynthesis is reduced (e.g., [56,57,58]). Comparing the course of photosynthesis (Figure 2A,B), we found that in the tolerant WIS, the net photosynthesis rate did not differ significantly between control and As treatments as opposed to sensitive SYL, where it dramatically decreased under As. The As-caused decrease of the photosynthesis rate was also reported in sensitive poplar [58] or chickpea [59], which may be related to thylakoid membrane damage. MDA accumulation indicates membrane damage that also involves chloroplast membranes. Thus, in sensitive SYL, higher MDA levels justify the observed lower photosynthetic efficiency. However, in this species, the photosynthesis disruption might also be connected with the decrease of chlorophyll a fluorescence appearing at the higher As dose (Figure 2E). In various plant species, heavy metal stresses have often been connected with a fluorescence decrease [60]. The decreasing proportion of active reaction centers under As treatment can reduce photochemical electron transport, resulting in a decrease of the maximum fluorescence efficiency of chlorophyll a [61]. In our study, the maximum fluorescence efficiency of the tolerant WIS was not influenced under As, in contrast to sensitive SYL, where a significant decrease appeared at 30 µg l^−1^ arsenate. Photosynthesis might also be disturbed by decreased plastid pigments contents, as shown by Mishra et al. [62] in rice, Malik et al. [59] in chickpea, and Naeem et al. [14,15] in *Artemisia annua*. Therefore, we also determined the contents of chlorophyll a and b and carotenoids (Figure 2C,D). With an increasing arsenic dose, the pigment contents in both genotypes decreased, but for SYL, the decrease was more prominent, and was significant even at 10 µg l^−1^ arsenate. The reduction in chlorophyll contents may have been caused by other factors, such as increased chlorophyllase activity or oxidative damage [63]. In this study, the impairment of the photosynthetic process induced by As was corroborated by lower sugar levels and the more severe growth reduction in sensitive SYL. The limitation of the photosynthesis rate may be a consequence of stomatal closure, which was found in rice [64]. We observed more affected stomatal conductance in sensitive SYL than in tolerant WIS (Figure S1). Together, we suppose that in SYL, the photosynthesis rate reflected stomatal conductivity limitations and a depletion of the photosynthetic pigments, resulting in a PSII efficiency decrease. Lowering of the photosynthesis rate in sensitive species is in accordance with the findings of Baker [65] or Liu et al. [58]. A decrease in the stomatal conductance could reduce water loss in As-treated plants. It has been reported that heavy metals affect water flow, thus disturbing the water status [66,67]. Changes in the water flow dynamics could be considered an adaptive strategy to regulate metal uptake and translocation, thus avoiding accumulation and toxicity. Nevertheless, a metal-induced reduction of water flow has been associated with a reduction in radial growth and changes in aquaporins, plasmodesmata, xylem, and stomata characteristics [68]. 

### 4.2. Arsenic-Induced ROS Accumulation 

Arsenic is not a redox metal; nevertheless, there is significant evidence that arsenic exposure does result in ROS generation in plants [69], involving singlet oxygen (^1^O_2_), superoxide radical (O_2_·^−^), hydrogen peroxide (H_2_O_2_), or hydroxyl radical (HO·) production. Cell damage caused by oxidative stress is considered to be a major adverse effect of heavy metal exposure [70]. Arsenic exposure led to H_2_O_2_ accumulation and cell damage by increased lipid peroxidation [25,71,72]. In this study, we hypothesized that the plants effectively coping with oxidative stress would thrive better under As treatment. Our results (Figure 3A,B) showed that the tolerant WIS exhibited a smaller increase of ROS than the sensitive SYL. Tolerant WIS under As stress had a minimum increase of MDA as opposed to the sensitive SYL, thus WIS’s membranes were less damaged (Figure 3C,D). Therefore, we can assume that the tolerant tobacco had a more effective antioxidant system than the sensitive one. To verify this assumption, we determined the antioxidant enzyme activities (Figure 4) of WIS and SYL plants. 

### 4.3. Antioxidant Enzyme Activities as Affected by As Stress

The enzymes activities varied greatly between genotypes as well as the organs analyzed. In the tolerant WIS under As, APX activity increased in roots and decreased in leaves. Sensitive SYL, on the other hand, reacted quite differently: Root activity was almost unchanged while leaf activity increased (Figure 4A,B). Similar trends were also found for POX with one exception: WIS roots, where the increase was not significant (Figure 4G,H). Interestingly, other enzyme activities also differed substantially between genotypes. Leaf GST activity exhibited reverse reactions: An increase in SYL and decrease in WIS with an increasing As dose. In roots, however, the activity did not change in WIS while it increased in SYL. It is possible, therefore, that the tolerant tobacco overcomes the ROS burst already in the roots by APX and POX activities while the sensitive tobacco induces these antioxidant enzyme systems as late as in leaves. Previous studies on the As effect have also shown changes in antioxidant enzymes activities [67]. Generally, plant As exposure mostly enhanced the activities of these enzymes [72] or at least some of them (e.g., [73]). The results of Gupta and Ahmad [74] also showed differential responses of the antioxidant enzymes (SOD, CAT, APX) in two contrasting rice varieties. They found higher activities in leaves of a tolerant variety. Cakmak [75] further proposed that an increase/decrease of CAT and APX activities could be due to substrate amounts’ increase or inactivation by protease. These enzymes are responsible for degradation of H_2_O_2_, a potential source of highly reactive (HO·) and (^1^O_2_). Thus, their higher activities are generally perceived as plant oxidative stress markers [74]. However, antioxidant enzymes cannot remove the most reactive ROS, (HO·). Therefore, determinations of antioxidant molecule levels, e.g., proline and soluble phenolic compounds or ascorbate, is necessary to create a more complete picture of the scavenging capacity. 

### 4.4. Antioxidant Molecules Accumulation Resulting from As Stress

Proline plays a protective role against ROS, e.g., protects membranes and proteins from inorganic ions’ adverse effects [76]. A study on rice [77] indicated an enhanced proline content with increasing concentrations of arsenic; however, in our As-treated plants, a statistically significant difference was found only in WIS leaves at the higher As concentration (Figure S2). The difference between our results and that of Saha et al. [77] could stand on species-specific reactions of proline upon metal stress as tobacco under cadmium also did not enhance leaf proline levels even after 25 days of treatment [78]. Phenolic compounds enhance antioxidant enzyme activities and serve as antioxidants themselves [55], probably also due to their readiness to chelate metals [27]. In our study, the phenolic compound contents tended to increase at both As levels in the leaves of both genotypes. In the roots, however, they significantly increased under both As treatments in WIS while SYL exhibited only a trend to increase (Figure 5B,C). We propose that phenolics substantially contributed to the defense mainly in WIS roots. Similarly, higher production of total phenolics and proline was found in the tolerant maize cultivar under arsenic treatment [79]. Among the important phenolic antioxidants anthocyanins belong [80], with the ability, besides free radical scavenging, to bind heavy metals and detoxify them in vacuoles [81,82]. The antioxidative properties of anthocyanins arise from their high reactivity as hydrogen or electron donors, from the ability of the polyphenol-derived radicals to stabilize and delocalize the unpaired electron, and from their ability to chelate transition metals [83]. In our study, the anthocyanins content exhibited stable levels in the tolerant WIS (Figure 5A) but dramatically decreased in the sensitive SYL at higher As. Anthocyanins are also an important sink of photosynthates, and in SYL, photosynthesis was disturbed at 30 µg l^−1^. In *Coriandrum,* a decrease of antioxidant contents, including anthocyanins, was found under As treatment, therefore *Coriandrum* is probably a sensitive plant [27]. In tolerant tomato, Kumar et al. [84] found increased anthocyanins accompanied by an increase in carotenoids. The authors proposed that through anthocyanins and carotenoids, tomato can effectively defend itself [84].

Ascorbate is another substance with the ability to improve plant growth and development due to its antioxidant property associated with plant resistance to oxidative damage [85] and is an integral part of the ascorbate–glutathione cycle. Both ascorbate and glutathione act in a very coordinated manner in this cycle and protect cells from oxidative disorders caused by H_2_O_2_ [19]. There was a marked significant increase in tolerant tobacco roots (Figure 5F). It is evident that this antioxidant contributes significantly to the antioxidant defense of tolerant tobacco. Increases in ascorbate content were observed, for example, in arsenic-exposed sunflower [15] or rapeseed [86]. These results are also consistent with an increase in APX activity in tolerant tobacco as APX utilizes the ascorbate-reducing force to decompose H_2_O_2_. Oppositely, in the roots of the sensitive genotype, ascorbate gradually decreased to about one third of the control in the 30 µg l^−1^ As treatment (Figure 5G). This decline was accompanied by a decrease in APX activity, which may be due to damage to this enzyme and other enzymes involved in ascorbate regeneration, such as monodehydroascorbate reductase and dehydroascorbate reductase [18].

It is known that glutathione plays a major role in protecting cells from arsenic [87] as it can directly detoxify ROS through GST activity and provide electrons for the reduction of As^V^ [88,89]. In this study we found a tendency to increase the total glutathione contents in the roots and leaves of both genotypes (Figure S3). However, the GSH:GSSG (reduced glutathione: glutathione disulphide) ratio is supposed to be more important than the total glutathione amount [90]. Under oxidative stress, the GSH:GSSG ratio usually decreases and this was also observed in our genotypes (Figure 5D,E), but more severe in WIS. GSSG accumulation presumably results from the reduction of GSSG by glutathione reductase (GR) or even damage of GR by arsenite binding. On the other hand, GSH can also be used for phytochelatins synthesis [91]. Similar results in glutathione metabolism were also found in rice under As treatment [92]. Interestingly, higher contents of GSSG were found in a tolerant genotype *Pteris vittata* under As than sensitive *Pteris ensiformis* [93]. Both genotypes had a lower GSH:GSSG ratio and the tolerant genotype even less. The authors explained that the tolerant *Pteris* species had a greater reduction power and lower GSH/GSSG ratio, which helps plants in several physiological functions, including activation/inactivation of redox-dependent enzymes and regeneration of the cellular antioxidants, e.g., ascorbic acid.

### 4.5. Carbohydrate Status Under As Stress

Protection against oxidative stress also includes the action of carbohydrates that can effectively quench free radicals [28,94]. Sugars, especially disaccharides (sucrose and trehalose), raffinose family oligosaccharides, and fructans participate in ROS elimination under abiotic stresses [95]. In addition, soluble sugars and starch play vital roles in osmotic regulation, membrane lipid biosynthesis, as sources of C and energy, and signal molecules [31]. The relationship between carbohydrate accumulation and enhanced heavy metal tolerance has been reported in some plant species, e.g., citruses [96] or rice [97]. The tolerant genotype WIS maintained similar levels of carbohydrates (soluble as well as starch) in the roots and leaves regardless of the As treatment (Figure 6), whereas the sensitive genotype exhibited a large decrease of sugar contents in the roots, though the root starch levels were not influenced. The leaf starch content showed a fluctuating trend in SYL: Starch increased under lower As but decreased under a higher As dose. Interestingly, in the roots of the tolerant genotype, there was a growing proportion of sucrose with an increasing As concentration (Figure 6A,B). Sucrose is the primary carbohydrate; thus, its metabolism is vital to the regulation of plant growth and stress responses [98]. As already mentioned, arsenic can bind to enzyme SH groups, which also applies, among others, to carbohydrate metabolism enzymes, and thereby can significantly affect their functions. Based on our results, we suggest that in the sensitive tobacco, carbohydrate metabolism is affected by arsenic treatment, both in the leaves and roots. This is in accordance with the results on relative As-sensitive tomato recently obtained by Gupta and Seth [16]. On the other hand, the carbohydrate levels of the tolerant genotype were not influenced, which is similar to As-tolerant *Pityrogramma calomelanos* [38]. Moreover, we observed that the tolerant tobacco grew relatively well even under high As stress, which we propose is a result of complex and efficient activation of a vast range of defense mechanisms, including enzymatic and non-enzymatic ROS quenching.

## 5. Conclusions

Study of the characteristics of tolerant versus sensitive plant genotypes can be fruitful for clarifying the strategies of the plant response in the face of oxidative stress caused by arsenic, as well as to evaluate the extent of the damage caused by an ROS level increase. In our study, we found that the growth of tolerant tobacco *N.tabacum*, cv. ’Wisconsin (WIS) was less affected by an arsenic compared to the sensitive *Nicotiana sylvestris* (SYL) genotype. We propose that this is the result of a complex metabolic response, including effective activation of the enzymatic antioxidant system connected with enhanced ascorbate peroxidase activity in the most exposed roots together with strengthening of the syntheses of antioxidant molecules, particularly ascorbate and phenolic compounds. Most importantly, the tolerant WIS exhibited less impaired photosynthetic processes, which allowed the carbohydrate balance to be maintained and provided C and energy, beside others, for antioxidant system activation, and probably directly involved sugars in ROS quenching. Taken together, we propose that the important innate features of As-tolerant plants are mainly an effective root-located antioxidant system, including both enzymatic and non-enzymatic components, and robust carbohydrate metabolism.

We further propose that future studies should concentrate on characterizing features of the photosynthetic apparatus or other physiological characteristics that are responsible for the low vulnerability of photosynthesis in As-tolerant plants. It will also be of great importance to find out whether the proposed conclusions are of general validity in plants or whether there is large variability in the mechanisms of tolerance in different plant species that have low susceptibility to As. Fulfillment of both the mentioned future tasks is indispensable for the creation of a reliable strategy for selection of plants suitable for phytoremediation as well as for breeding of As-tolerant crops.

## Supplementary Materials

The following are available online at https://www.mdpi.com/2076-3921/9/4/283/s1, Figure S1: Effects of arsenic on stomatal conductance in two contrasting tobacco genotypes, Figure S2: Effects of arsenic on proline contents in two contrasting tobacco genotypes, Figure S3: Effects of arsenic on total glutathione contents in two contrasting tobacco genotypes.
