# Supplementary material for: Multi-Component Antioxidative System and Robust Carbohydrate Status, the Essence of Plant Arsenic Tolerance
Source: Antioxidants (Basel). 2020 Mar 27;9(4):283. doi: 10.3390/antiox9040283 (PMC7222215; doi:10.3390/antiox9040283)
Supplement: Supplementary file 1 [file antioxidants-09-00283-s001.pdf]

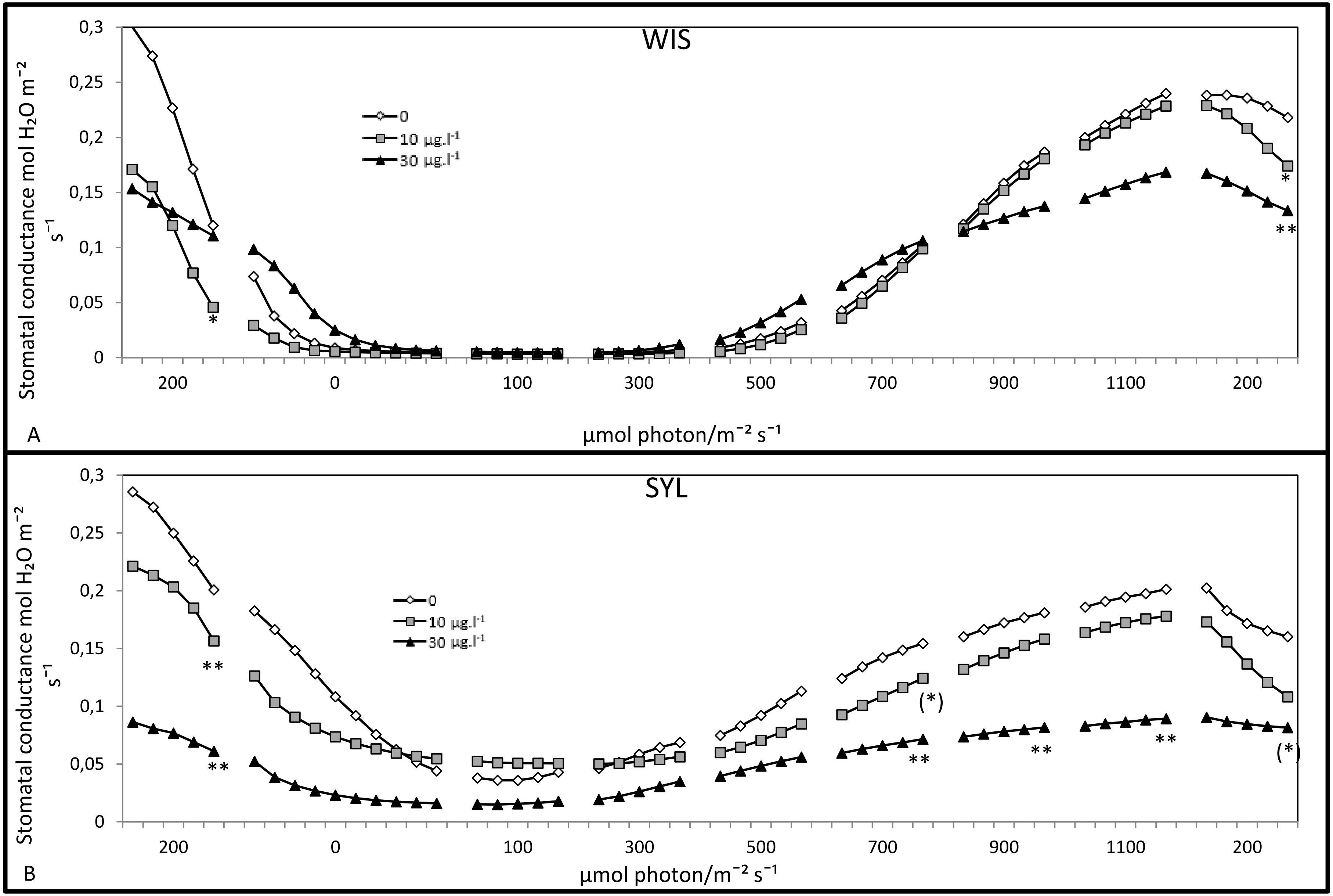

Fig.S1: Effects of arsenic on stomatal conductance in two contrasting tobacco genotypes (A, C – tolerant *N.tabacum* Wisconsin, B, D – sensitive *N. sylvestris*). 0, 10, and 30 µg.l^{-1} - arsenic treatments. Bars indicate standard deviations. Stars mean a statistically significant differences from control at the level α = 0.1 ((*)), 0.05(*), 0.01 (**) or 0.001(***), n = 5-7

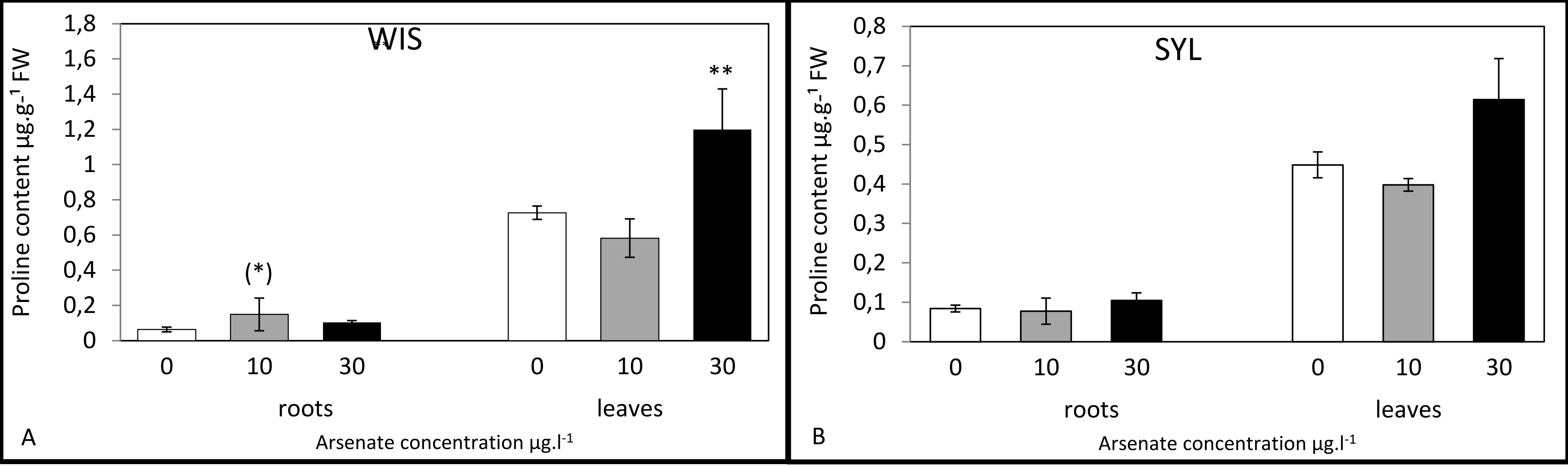

Fig.S2: Effects of arsenic on proline contents in two contrasting tobacco genotypes (A, C – tolerant *N.tabacum* Wisconsin, B, D – sensitive *N. sylvestris*). 0, 10, and 30 µg.l^{-1} - arsenic treatments. Bars indicate standard deviations. Stars mean a statistically significant differences from control at the level α = 0.1 ((*)), 0.05(*), 0.01 (**) or 0.001(***), n = 5-7

Proline level was measured following Bates (1973). Samples (0.5 g) were crushed in 2 ml of 3% sulfosalicylic acid (v/v) and centrifuged at 4472 x g for 5 min at room temperature. Whole supernatant sample was mixed with equivalent volume of ninhydrin and concentrated acetic acid and incubated at boiling water for 1h. The proline was extracted with 3 ml of toluene and quantified spectrophotometrically at 520 nm.

Bates, L.S. SHORT COMMUNICATION Rapid determination of free proline for w a t e r - s t r e s s studies. Plant and Soil 1973, 39, 205-207.

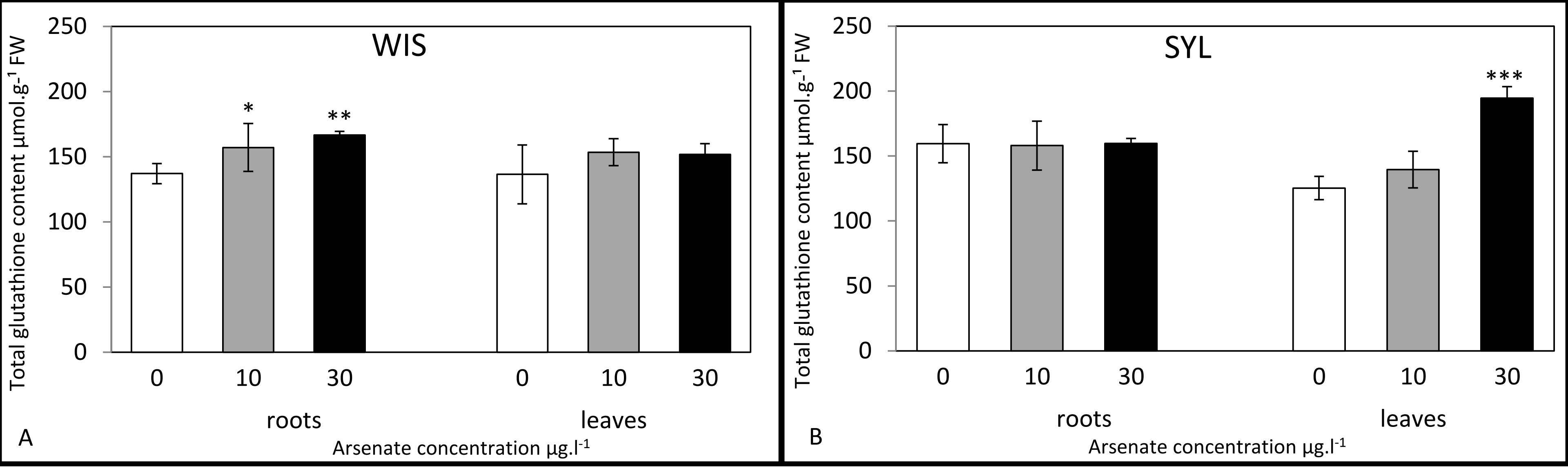

Fig.S3: Effects of arsenic on total glutathione contents in two contrasting tobacco genotypes (A, C – tolerant *N.tabacum* Wisconsin, B, D – sensitive *N. sylvestris*). 0, 10, and 30 µg.l^{-1} - arsenic treatments. Bars indicate standard deviations. Stars mean a statistically significant differences from control at the level α = 0.1 ((*)), 0.05(*), 0.01 (**) or 0.001(***), n = 5-7
